# The acute effects of moderate-intensity continuous or high-intensity interval exercise on appetite and appetite-related hormones in South Asian and white European adults with non-diabetic hyperglycaemia

**DOI:** 10.1038/s41430-025-01633-x

**Published:** 2025-05-27

**Authors:** Tonghui Shen, Alice E. Thackray, Jack A. Sargeant, Thomas Yates, James A. King, Scott A. Willis, David J. Stensel

**Affiliations:** 1 Department of Physical Education and Aesthetic Education, Hangzhou City University, Zhejiang, China; 2 National Centre for Sport and Exercise Medicine, School of Sport, Exercise and Health Sciences, Loughborough University, Loughborough, United Kingdom; 3 National Institute for Health and Care Research (NIHR) Leicester Biomedical Research Centre, University Hospitals of Leicester NHS Trust and University of Leicester, Leicester, United Kingdom; 4 Diabetes Research Centre, University of Leicester, Leicester, United Kingdom; 5 Faculty of Sport Sciences, Waseda University, Tokorozawa, Japan; 6 Department of Sports Science and Physical Education, The Chinese University of Hong Kong, Hong Kong, China

**Keywords:** Risk factors, Weight management, Biomarkers

## Abstract

**Objective:**

To compare acute effects of continuous moderate-intensity exercise (CME) and low-volume high-intensity interval exercise (LV-HIIE) on appetite responses between South Asians and white Europeans with non-diabetic hyperglycaemia.

**Methods:**

Thirteen white Europeans and 10 South Asians (age 50–74 years) completed three, 5-h experimental conditions (CME, LV-HIIE, control) in randomised sequences. Standardised meals were provided at 0 and 3 h. Exercise involved a 25-min LV-HIIE or 35-min CME bout that ended at 2 h. Subjective appetite perceptions and appetite-related hormones (glucagon-like peptide-1 (GLP-1), peptide YY (PYY), acylated ghrelin (AG)) were measured at 0, 0.5, 1, 2, 3, 3.5, 4 and 5 h. Time-averaged total area under the curve (TAUC; 1–5 h) was analysed, adjusted for age, sex, and pre-intervention time-averaged TAUC (0–1 h).

**Results:**

Total GLP-1 was higher in LV-HIIE (mean difference [95% CI] 4.3 [1.5, 7.1] pmol/L h) and CME (4.5 [1.4, 7.7] pmol/L h) versus control (condition effect *P* = 0.01), but exercise had no effect on the other outcomes in the whole study population (*P* ≥ 0.28). Appetite responses to exercise were similar between ethnicities for total GLP-1 and total PYY (interaction *P* ≥ 0.11), but subtle differences emerged for AG and overall appetite (interaction *P* ≤ 0.02). AG was higher and overall appetite lower in LV-HIIE versus CME in South Asians whilst overall appetite was higher in LV-HIIE versus CME in white Europeans, but neither exercise bout was different to control.

**Discussion:**

Single LV-HIIE and CME bouts increased total GLP-1 in individuals with non-diabetic hyperglycaemia, but exercise-related appetite responses were not strongly modulated by exercise intensity or ethnicity.

## Introduction

South Asians constitute the largest ethnic minority in the UK [1] and possess a substantially increased risk of type 2 diabetes and cardiovascular disease compared with white Europeans [2]. These conditions typically develop 5–10 years earlier in South Asians and at a lower adiposity [3]. This heightened risk is accompanied by a more adverse cardiometabolic profile in South Asians including greater insulin resistance, hyperglycaemia and dyslipidaemia [4] and has been partly attributed to differences in adiposity between ethnicities [5, 6]. South Asians exhibit a higher proportion of body fat, particularly in central regions, and less lean tissue for any given body mass index (BMI) [5, 6]. Consequently, ethnic-specific BMI cut-offs have been developed for South Asians to define overweight and obesity [7].

These ethnic differences in adiposity and cardiometabolic risk may be associated with the differential regulation of appetite and appetite-related hormones [8]. Fasting concentrations of acylated ghrelin (AG), an orexigenic hormone, were recently observed to be lower in South Asian men compared to BMI-matched white European men [8]. Additionally, other studies have reported that individuals with type 2 diabetes and/or obesity exhibit lower fasting AG concentrations than lean individuals [9, 10] along with a blunted elevation of postprandial anorexigenic hormone concentrations including peptide YY (PYY) and glucagon-like peptide-1 (GLP-1) [11–13].

Single bouts of exercise suppress appetite and AG while increasing GLP-1 and PYY concentrations [14, 15]. However, comparisons between ethnicities are scarce with the only study to date demonstrating that healthy South Asian and white European men exhibited comparable increases in circulating total PYY and decreases in circulating AG after 60 min of moderate-to-vigorous intensity cycling; total GLP-1 was not measured [16]. Exercise-related responses may differ according to adiposity status with individuals with overweight/obesity experiencing a greater elevation in total GLP-1 but a blunted increase in total PYY in response to acute exercise when compared to lean individuals [17]. Limited exercise-related research exists in those with impaired fasting glucose, impaired glucose tolerance or diagnosed type 2 diabetes [18] but given the prominent predisposition of South Asians to overweight/obesity and related cardiometabolic disease, further ethnic comparisons are warranted in populations with these conditions.

Exercise intensity has also been proposed to modulate the appetite-related hormone response to acute exercise [19, 20]; however, findings remain conflicting. In healthy, lean individuals, high-intensity interval exercise (HIIE) (ten, 4-min cycling intervals at 85% V̇O_2_ max) elicited greater increases in circulating PYY compared to isoenergetic continuous moderate-intensity exercise (CME, 60 min cycling at 60% V̇O_2_ max) [21]; whilst circulating AG concentrations were suppressed to a greater extent after sprint interval exercise (six, 30-s maximal sprints with 4.5-min active recovery periods) versus CME [22]. Conversely, in individuals with overweight or obesity, HIIE protocols appear to produce similar responses in suppressing AG and subjective appetite perceptions whilst elevating GLP-1 and PYY compared to CME [23–25]. Whether South Asian and white European individuals respond differently to acute HIIE versus CME is yet to be explored.

This study sought to compare subjective appetite perception and appetite-related hormone (total GLP-1, total PYY, AG) responses to low-volume (LV)-HIIE and CME in white European and South Asian individuals with non-diabetic hyperglycaemia. We hypothesised that circulating concentrations of total GLP-1 and total PYY would be increased after each exercise bout, whilst overall appetite and circulating AG would be suppressed. Furthermore, these responses would be more pronounced in South Asians compared with white Europeans.

## Methods

### Ethical approval and participant recruitment

This manuscript presents secondary outcomes from a published randomised, crossover study investigating ethnic differences in circulating metabolic responses to acute exercise bouts of differing intensities [26]. The study was approved by an NHS research ethics committee (15/EM/0259) and registered as a clinical trial (ISRCTN12337078). All methods were performed in accordance with the relevant guidelines and regulations and written informed consent was obtained from all participants.

Men and postmenopausal women of white European or South Asian descent were recruited if they were aged 50–74 years, had a BMI ≥27.5 kg/m^2^ if white European or ≥25.0 kg/m^2^ if South Asian, were weight stable (< 5 kg change over the last 6 months), and not excessively physically active (<3 self-reported vigorous-intensity exercise sessions per week [≥20 min per session]). South Asian ethnicity was defined as anyone identifying as “Asian” or “Asian British (Indian, Pakistani, Bangladeshi)”, and white Europeans identified as “white/Caucasian” and descended from any European country. Participants had non-diabetic hyperglycaemia (glycated haemoglobin (HbA1c) 39–47 mmol/mol or 2-h plasma glucose 7.8–11.0 mmol/L during a 75 g oral glucose tolerance test) but were free of diagnosed chronic metabolic disease and had no contraindications to any study procedure.

### Experimental design

**Fig. 1:**
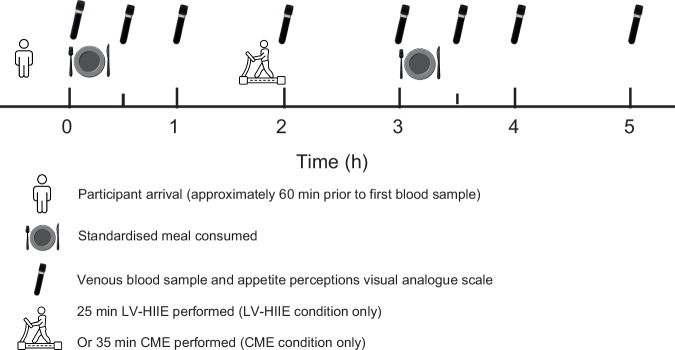
Schematic of the experimental trials. LV-HIIE low volume-high-intensity interval exercise, CME continuous moderate-intensity aerobic exercise.

The experimental design and study procedures have been described in detail previously [26]. Briefly, after a preliminary familiarisation visit, participants completed three 6-h experimental conditions (LV-HIIE, CME and sedentary control [CON]) in a randomised, crossover design separated by approximately 7 days. Conditions were completed in a randomised order stratified by sex and ethnicity. The outcomes reported in this study were calculated from 0 to 5 h and the study design is presented schematically in Fig. 1.

### Preliminary visit

Participants attended a preliminary visit in the non-fasted state and underwent medical evaluation with blood screen and cardiovascular risk assessment to confirm eligibility [26]. Measurements of body mass (Tanita TBE 611, Tanita, West Drayton, UK), height (Leicester Height Scale, Seca, Birmingham, UK) and waist circumference were taken. BMI (kg/m^2^) was subsequently calculated.

Participants then undertook an incremental maximal exercise test on a motorised treadmill (Woodway PPS 70 Plus, Woodway USA Inc., USA) under the supervision of a specialist cardiac nurse to determine their V̇O_2_ peak. After a 3-minute warm-up, participants walked briskly at a self-selected speed and 0% incline which increased by 1% every minute until volitional exhaustion. Expired air was sampled continuously using an online breath-by-breath gas analysis system (Metalyser 3B, Cortex Biophysik GmbH, Germany), and heart rate was monitored continuously throughout. The exercise testing was aborted by a cardiac nurse and participation ceased for two participants who presented with adverse symptoms (*n* = 1 white European female, chest pain and breathlessness; *n* = 1 South Asian female, severe ankle pain) as described previously [26]. In the absence of clinical indications, the test continued until volitional exhaustion or until participants reached both 100% of their age-predicted maximum heart rate and a respiratory exchange ratio ≥1.15. After at least 15 min recovery, participants completed a short familiarisation of the LV-HIIE protocol consisting of three intervals.

### Experimental visits

After a 10-h overnight fast, participants arrived at the laboratory at ~08:00 and an intravenous cannula (Braun, Pennine Healthcare, UK) was inserted into an antecubital vein by a trained phlebotomist. After 30–60 min of habituation, a fasting venous blood sample (0 h) was drawn and subsequent blood samples were collected at 0.5, 1, 2, 3, 3.5, 4 and 5 h for the determination of AG, total PYY, total GLP-1 and leptin (fasting only) concentrations. Identical, standardised meals were provided at 0 and 3 h and the meal quantity was scaled to baseline body mass (approximately 8 kcal energy per kilogram of body mass). Due to variation in food preferences and dietary requirements, meals consisted of a white bagel and full-fat margarine with either (option 1) full-fat cheddar cheese, fruit jelly, and orange juice or (option 2) a meal-replacement shake made with whole-milk. The meal was expected to induce a sufficient metabolic challenge for assessing the primary study outcomes related to glucose and lipid metabolism [26], and participants consumed the same meal during their respective experimental conditions.

In the exercise trials, participants undertook either a 25-min LV-HIIE protocol or a 35-min CME protocol at a staggered start time in order for exercise to be completed at 2 h. LV-HIIE comprised of 10 × 1-min intervals of brisk walking at the same self-selected treadmill speed chosen during the preliminary visit familiarisation and a predicted incline to elicit 90% of V̇O_2_ peak. The intervals were interspersed with 1 min of walking recovery at 3.5 km/h and 0% incline. CME involved 30 minutes of continuous brisk walking at the same self-selected treadmill speed as the preliminary visit familiarisation and a predicted incline to elicit 65% of V̇O_2_ peak. Both exercise protocols also included an identical 3-minute warm-up and 2-min cool-down period at 3.5 km/h and 0% incline. Participants were instructed to remain rested in a seated position for the remainder of the LV-HIIE and CME trials, and for the entirety of the CON trial (no exercise performed). Compliance to this instruction was confirmed through a thigh-worn accelerometer (activPAL, PAL Technologies, Glasgow, UK) which monitored time spent sitting and standing throughout each trial (data published previously [26]).

### Appetite perceptions

Subjective appetite perceptions (hunger, satisfaction, fullness, prospective food consumption) were assessed immediately before the blood sample collection using 100 mm visual analogue scales [27]. An overall appetite rating was calculated as the mean value of the four appetite perceptions after reversing the values for satisfaction and fullness [28].

### Biochemical analysis

HbA1c was analysed using standardized quality-controlled enzymatic assays by the clinical pathology laboratories of University Hospitals of Leicester NHS Trust. Blood samples were drawn into pre-chilled EDTA monovettes (Sarstedt, Leicester, UK) and tubes for AG also contained a protease inhibitor cocktail in line with the manufacturer’s guidelines. Monovettes were centrifuged immediately at 4 °C before storage at −80 °C for later batch analysis. Commercially available enzyme-linked immunosorbent assays were used to measure plasma concentrations of total GLP-1 (Merck Millipore, Darmstadt, Germany; CV_intra_ 3.48%), total PYY (Merck Millipore, Darmstadt, Germany; CV_intra_ 7.45%), AG (Bertin Technologies, France; CV_intra_ 6.22%) and leptin (R&D Systems, Oxford, UK; CV_intra_ 1.65%).

### Statistical analyses

Given that the data reported in this manuscript are secondary outcomes from a previous trial [26], the present analysis was not informed by a formal power calculation. Data were analysed using the IBM SPSS Statistics software package for Windows version 25.0 (IBM Corporation, New York, USA). To examine appetite responses between meal options 1 and 2, generalised linear models were performed with an identity link function and gamma distribution, adjusting for age, sex and ethnicity. This analysis used the time-averaged (0–5 h) incremental area under the curve (iAUC) for the control trial, calculated using the trapezium rule with baseline defined as the fasted (total GLP-1, total PYY) or nadir (acylated ghrelin, overall appetite) value.

For the main analysis, time-averaged total (T)AUC was calculated for all outcomes during the pre-intervention (0–1 h) and intervention (1–5 h) periods. Time-averaged TAUC (1–5 h) for each study outcome were compared between trials and ethnicities using generalised estimating equations specifying a gamma distribution (positively skewed data) and exchangeable correlation matrix. Models for appetite-related hormones used an identity link function whereas overall appetite was modelled with a log link function based on the best model fit. All models contained an interaction term between ethnicity and condition and were adjusted for age, sex, and pre-intervention time-averaged TAUC (0–1 h). To aid interpretation, outcomes across each condition are reported stratified by ethnicity and for the combined study population.

Missing data for study outcomes were imputed using a regression model reported previously [29], with age, sex, ethnicity, BMI, HbA1c, fasting values and experimental condition as predictors. Imputations were performed for 9.1%, 7.6%, 7.4% and 1.6% of the total PYY, total GLP-1, AG and overall appetite datapoints, respectively. The 95% confidence intervals (CIs) were calculated for mean pairwise differences between conditions and ethnic groups. Absolute standardised effect sizes (Cohen’s *d*) were calculated by dividing the mean difference by the pooled SD. Thresholds of 0.2, 0.5 and 0.8 were used to define small, medium and large effects, respectively. *P* < 0.05 was considered statistically significant.

## Results

### Participant characteristics

The participant recruitment process for this study has been reported previously [26]. Overall, 23 participants (13 white Europeans, 10 South Asians) completed the study and the baseline characteristics of the cohort stratified by ethnicity are presented in Table 1. The white European group were older with a greater proportion of female participants and had a lower prevalence of obesity compared to the South Asian group.

**Table 1:** Participant characteristics.

	All (*n* = 23)	White European (*n* = 13)	South Asian (*n* = 10)
Sex (M/F)	13/10	6/7	7/3
*Anthropometry*			
Age (years)	64 (7)	68 (3)	60 (7)
Body mass (kg)	82.6 (11.6)	83.7 (10.2)	81.3 (13.7)
BMI (kg/m^2^)	30.5 (3.5)	31.1 (4.0)	29.8 (2.8)
Waist circumference (cm)^a^	102.2 (8.6)	102.8 (7.2)	101.3 (10.6)
Obesity prevalence, *n* (%)^b^	13 (57)	6 (46)	7 (70)
*Cardiorespiratory fitness*			
Absolute V̇O_2_ peak (L/min)	3.4 (0.8)	3.3 (0.6)	3.5 (1.0)
Relative V̇O_2_ peak (mL/kg/min)	25.2 (4.5)	25.7 (4.5)	24.7 (4.6)
*Fasted appetite perceptions and appetite-related hormones*			
Overall appetite (mm)	65 (15)	61 (13)	70 (15)
Total GLP-1 (pmol/L)	44.3 (12.8)	43.9 (13.5)	44.7 (12.5)
Total PYY (pg/mL)	85.9 (54.6)	79.7 (41.0)	94.0 (70.2)
Acylated ghrelin (pg/mL)	69.4 (44.0)	76.8 (46.2)	59.9 (41.4)
Leptin (pg/mL)	22,493 (13,574)	21,666 (14,199)	23,568 (13,390)

All values are mean (SD) unless otherwise indicated.

^a^Data for 1 South Asian female missing so data presented for *n* = 22.

^b^Ethnicity-specific BMI thresholds were used to categorise obesity prevalence (BMI ≥ 30.0 kg/m^2^ and ≥27.5 kg/m^2^ in white European and South Asian groups, respectively).

### Standardised meals

The mean energy content of the meals was 663 (128) kcal (range 376–997 kcal) with a relative contribution of 62(2)% carbohydrate, 21(2)% fat and 17(1)% protein. Among the South Asian group, seven consumed meal option 1 and three consumed meal option 2, whereas all white Europeans consumed meal option 2. The energy content of the two meals was similar (7.8 vs 8.0 kcal/kg body mass for meal 1 and 2, respectively; *P* = 0.853). The only difference in macronutrient composition was a lower protein contribution in meal 1 compared to meal 2 (15.3% vs 17.3% of total energy, respectively; mean difference [95% CI] −2.0 [−3.8 to −0.1]%, *P* = 0.040). There were no statistically significant differences in time-averaged iAUC between meals 1 and 2 for any appetite outcome (all *P* ≥ 0.260).

### Appetite-related outcomes

**Fig. 2:**
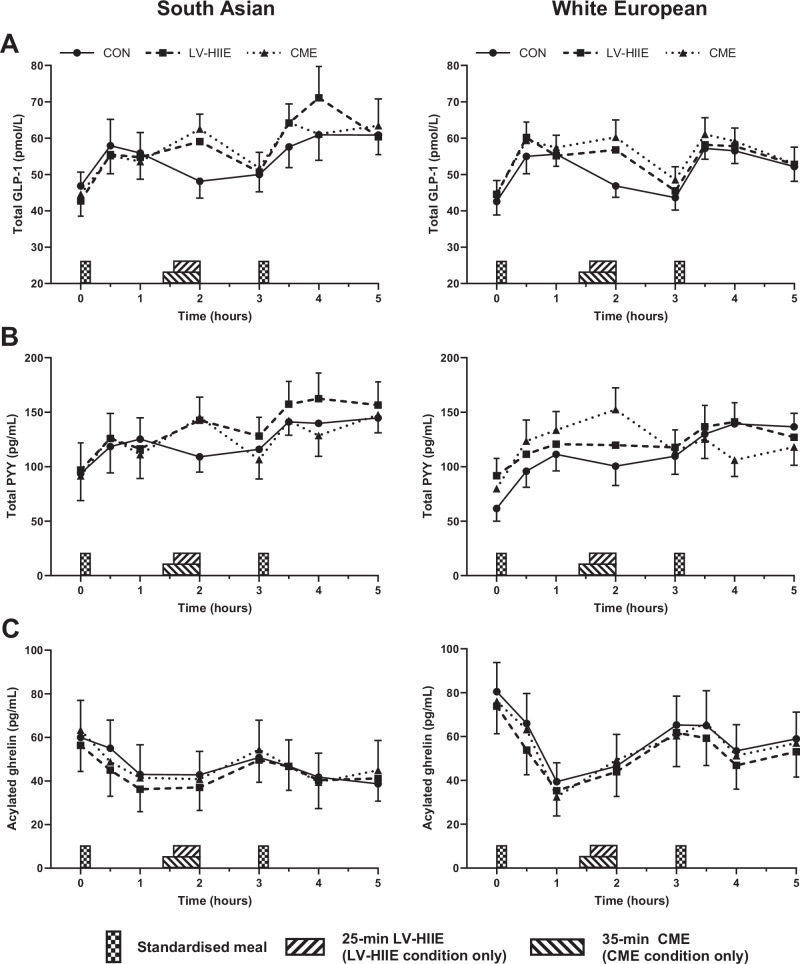
Plasma concentrations of appetite-related hormones across experimental conditions for South Asians and white Europeans. Data presented for total GLP-1 (**A**, top panels), total PYY (**B**, middle panels) and acylated ghrelin (**C**, bottom panels). Data are mean and standard error of the mean for *n* = 23 (10 South Asians and 13 white Europeans) apart from total PYY which is presented for *n* = 22 (10 South Asians and 12 white Europeans). CON seated, rested control condition, LV-HIIE low-volume high-intensity interval exercise, CME continuous moderate-intensity aerobic exercise, GLP-1 glucagon-like peptide-1, PYY peptide YY.

Analysis of the time-averaged TAUC for total GLP-1 revealed a main effect of condition (*P* = 0.01), but no main effect of ethnicity (*P* = 0.48) or condition-by-ethnicity interaction (*P* = 0.13) (Table 2, Fig. 2). In the combined study population, time-averaged TAUC for total GLP-1 was higher in both exercise conditions compared to CON by 4.3 (95% CI 1.5–7.1) pmol/L h (*d* = 0.65; *P* = 0.003) and 4.5 (95% CI 1.4–7.7) pmol/L h (*d* = 0.75; *P* = 0.01) in LV-HIIE and CME, respectively (Table 3).

**Table 2:** Time-averaged TAUC (1–5 h) for overall appetite perceptions and appetite-related hormones during each condition for each ethnic group.

	White European			South Asian			*P* value		
	CON	LV-HIIE	CME	CON	LV-HIIE	CME	*Cnd*	*Eth*	*Int*
Overall appetite (mm h)	35 (31, 40)	38 (33, 44)^*^	33 (29, 38)^*^	37 (32, 43)	32 (25, 40)^*^	39 (32, 48)^*^	0.82	0.88	**0.02**
Total GLP-1 (pmol/L h)	53.1 (49.6, 56.6)	54.6 (50.7, 58.5)	55.5 (50.9, 60.2)	52.3 (47.1, 57.5)	59.4 (53.9, 64.9)	58.9 (54.5, 63.4)	**0.01**	0.48	0.13
Total PYY (pg/mL h)	136 (125, 147)	125 (115, 136)	124 (115, 133)	131 (118, 143)	134 (121, 147)	127 (113, 141)	0.28	0.77	0.11
Acylated ghrelin (pg/mL h)	46.0 (40.9, 51.2)	45.6 (40.0, 51.2)	46.7 (40.8, 52.6)	46.5 (41.3, 51.7)	49.2 (43.0, 55.4)^*^	45.8 (40.1, 51.6)^*^	0.34	0.32	**0.02**

Time-averaged TAUC (1–5 h) data are presented as geometric mean (95% CI) for overall appetite and as mean (95% CI) for appetite-related hormones. Values displayed for *n* = 23 (13 white Europeans and 10 South Asians) apart from total PYY which is presented for *n* = 22 (12 white Europeans and 10 South Asians). All models were adjusted for age, sex and pre-intervention TAUC (0–1 h).

*Cnd* main effect of condition, *Eth* main effect of ethnicity, *Int* condition-by-ethnicity interaction, *CON* seated, rested control condition, *LV-HIIE* low-volume high-intensity interval exercise, *CME* continuous moderate-intensity aerobic exercise, *GLP-1* glucagon-like peptide-1, *PYY* peptide YY.

^*^P ≤ 0.05 between exercise trials within ethnic group. *P* values shown in bold indicate the main or interaction effect was statistically significant (*P* < 0.05). Data for each condition in the combined population can be found in Table 3.

**Table 3:** Time-averaged TAUC (1–5 h) for overall appetite perceptions and appetite-related hormones during each condition in the combined study population.

	CON	LV-HIIE	CME
Overall appetite (mm h)	36 (33, 40)	35 (30, 40)	36 (32, 41)
Total GLP-1 (pmol/L h)	52.7 (50.2, 55.2)	57.0 (54.2, 59.8)^*^	57.2 (54.8, 59.7)^*^
Total PYY (pg/mL h)	133 (125, 142)	130 (121, 138)	125 (117, 133)
Acylated ghrelin (pg/mL h)	46.3 (41.3, 51.2)	47.4 (41.7, 53.1)	46.3 (40.6, 51.9)

Time-averaged TAUC (1–5 h) data are presented as geometric mean (95% CI) for overall appetite and as mean (95% CI) for appetite-related hormones. Values displayed for *n* = 23 (13 white Europeans and 10 South Asians) apart from total PYY which is presented for *n* = 22 (12 white Europeans and 10 South Asians). All models were adjusted for age, sex and pre-intervention TAUC (0–1 h).

*CON* seated, rested control condition, *LV-HIIE* low-volume high-intensity interval exercise, *CME* continuous moderate-intensity aerobic exercise, *GLP-1* glucagon-like peptide 1, *PYY* peptide YY.

^*^P ≤ 0.05 vs. CON.

A condition-by-ethnicity interaction (*P* = 0.02) was identified for time-averaged TAUC for AG but no main effect of condition nor ethnicity (*P* ≥ 0.32) (Tables 2 and 3, Fig. 2). Time-averaged TAUC for AG was higher in LV-HIIE compared to CME in the South Asian group (mean difference [95% CI]: 3.4 [0.8–6.0] pg/mL h; *d* = 0.23; *P* = 0.01) but no between-condition differences were apparent in the white European group (*P* ≥ 0.23) (Table 2, Fig. 2).

Time-averaged TAUC for total PYY was similar across conditions and ethnic groups and there was no condition-by-ethnicity interaction (*P* ≥ 0.11) (Tables 2 and 3, Fig. 2).

**Fig. 3:**
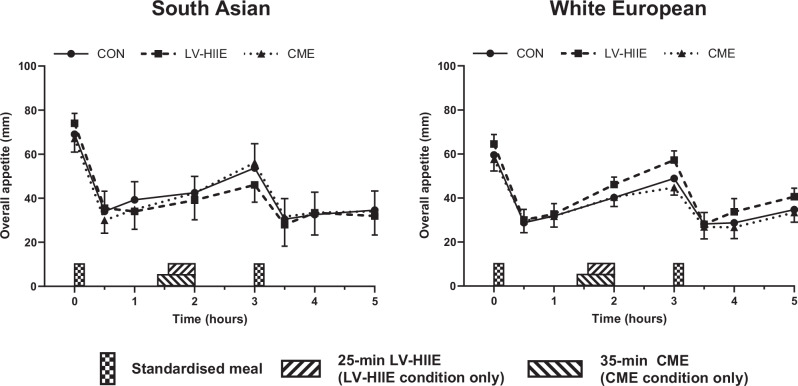
Overall appetite perceptions across experimental conditions for South Asians and white Europeans. Data are mean and standard error of the mean for *n* = 23 (10 South Asians and 13 white Europeans). CON seated, rested control condition, LV-HIIE low-volume high-intensity interval exercise, CME continuous moderate-intensity aerobic exercise.

Analysis of the time-averaged TAUC for overall appetite identified a condition-by-ethnicity interaction (*P* = 0.02) but no main effect of condition or ethnicity (*P* ≥ 0.82) (Tables 2 and 3, Fig. 3). Time-averaged TAUC for overall appetite was lower in LV-HIIE compared to CME in South Asians (geometric mean difference [95% CI]: −8 [−15 to 0] mm h; *d* = 0.39; *P* = 0.05) but was higher in LV-HIIE compared to CME in the white European group (geometric mean difference [95% CI]: 5 [0–10] mm h; *d* = 0.42; *P* = 0.05) (Table 2, Fig. 3).

## Discussion

This analysis examined whether the effect of single LV-HIIE or CME bouts on hormonal and subjective appetite-related responses differ between white European and South Asian adults with non-diabetic hyperglycaemia. The primary findings of this analysis were that total GLP-1 was increased in response to both LV-HIIE and CME compared to control, whilst acute exercise had no effect on overall appetite, total PYY, and AG in the whole study population. Furthermore, the appetite-related responses to acute exercise relative to control were largely similar between the ethnic groups and not supportive of our a priori hypothesis.

Both LV-HIIE and CME increased time-averaged TAUC for total GLP-1 to a similar extent in our population with non-diabetic hyperglycaemia. This finding extends previous investigations demonstrating that postprandial exercise increases circulating total GLP-1 independent of exercise intensity in lean individuals [30, 31] and individuals with overweight and obesity [23, 24]. Notably, our analyses are the first to examine ethnic differences in the total GLP-1 response to acute exercise between white Europeans and South Asians. Although no main ethnicity effect or condition-by-ethnicity interaction was observed, the exercise-related increase in total GLP-1 visually appeared more pronounced in the South Asian group, particularly after the meal consumed at 3 h (Fig. 2A). Our previous data showed that South Asians had greater reductions in circulating insulin and the insulin resistance index after LV-HIIE and CME compared to white Europeans [26]. Given insulin resistance has been associated with impaired postprandial satiety signalling and incretin responses (particularly GLP-1) [32], short-term improvements in insulin sensitivity could contribute to the potentially subtle augmented exercise-related total GLP-1 responses in South Asians. However, additional mechanistic experiments with a larger sample size are required to investigate this hypothesis directly.

Consistent with previous research in individuals with overweight and obesity [23, 33–36], and healthy South Asian and white European men (mean BMI 25 kg/m^2^) [16], LV-HIIE and CME did not alter subjective appetite perceptions in the combined study population. In contrast, a suppression in appetite is usually observed in response to single exercise bouts in individuals with healthy body weight (BMI < 25 kg/m^2^) [15, 17]. Most studies typically involve performing exercise after an overnight fast when pre-exercise appetite perceptions are higher than those recorded in this study commencing after breakfast consumption (376–997 kcal) and, therefore, the potential for exercise to suppress appetite may be greater [17, 37, 38]. It is also possible that a greater exercise intensity and/or volume may be required before clear appetite effects, including potential ethnic differences, are realised particularly given brisk walking protocols often demonstrate little influence on appetite [39, 40]. Interestingly, however, a condition-by-ethnicity interaction indicated that the South Asians exhibited a small suppression of overall appetite after LV-HIIE versus CME whereas the opposing relationship was evident in white Europeans. The reason for this disparity is unclear and, for the white European group, appears to contrast evidence showing similar if not greater appetite suppression when exercise is performed at higher intensities [21, 22, 41]. In the South Asians, pre-exercise overall appetite was higher in LV-HIIE versus CME which may increase the potential for appetite suppression, or alternatively the stimulus provided by LV-HIIE could have been perceived more potently given the South Asian individuals were less accustomed to more intense physical activity [26].

Moderate-to-vigorous intensity exercise has been shown to increase total PYY concentrations to a similar extent in healthy South Asians and white Europeans [16]. Whilst we similarly observed no differences between ethnic groups, circulating total PYY concentrations were unaffected by acute bouts of exercise in our cohort which appears to contrast the consensus of evidence demonstrating exercise-induced elevations in PYY concentrations [14, 15]. The greater levels of adiposity in the present study could be a contributing factor as the circulating total PYY response to acute exercise is reported to be trivial [42] or blunted [17] in populations with overweight and obesity. Additionally, compared to individuals with normal glucose regulation, an attenuated postprandial total PYY response to meal intake has been reported in individuals with impaired glycaemic control, insulin resistance or genetic susceptibility to type 2 diabetes [12, 43]. Therefore, higher levels of adiposity and/or insulin resistance may be acting to negate exercise-induced changes in circulating total PYY in our study population with non-diabetic hyperglycaemia.

Previous meta-analyses have demonstrated that acute exercise invokes a small-to-moderate suppression of circulating AG concentrations in both lean individuals and individuals with overweight and obesity [14, 42]. Furthermore, moderate-to-vigorous intensity cycling has been shown to suppress AG concentrations by a similar magnitude in healthy South Asians and white Europeans [16]. Our data are conflicting with these findings as the time-averaged TAUC for AG was not different to CON after LV-HIIE or CME in both ethnic groups. Exercise intensity has been proposed as an important determinant of the AG response to exercise [19, 20] with prolonged high-intensity exercise suggested to be most effective for suppressing ghrelin concentrations [44]. Although HIIE and LV-HIIE protocols (running or cycling) have been shown to suppress acylated [23, 45] and total [25] ghrelin, it appears the intensity of walking exercise in the present study may provide an insufficient physiological challenge to alter circulating AG concentrations. In support, other studies employing walking exercise protocols (40–60 min at 45–50% V̇O_2_ peak or 65–75% age-predicted maximal heart rate) have also failed to observe a suppression of AG concentrations [34, 36, 39, 40]. Low pre-exercise AG concentrations may also be a contributing factor in this study resulting from postprandial suppression after the standardised breakfast meal and/or the presence of overweight/obesity and insulin resistance in the study population.

Strengths of the current study include the randomised crossover trial design, strict standardisation procedures and novel research focus involving ethnic comparisons of different exercise intensities in an older population with non-diabetic hyperglycaemia. However, certain limitations must also be recognised. Some differences were evident in participant characteristics between the ethnic groups particularly in age and sex distribution. Although all statistical models were adjusted for age and sex, it is possible that other between-group differences could influence the findings. Expired air samples were not measured during the exercise bouts which precluded precise quantification of the exercise intensity and exercise-induced energy expenditure; therefore, comparisons are difficult with other studies employing similar exercise protocols or with different types of exercise. Additionally, post-intervention meals were not provided *ad libitum* limiting the ability to examine ethnic differences in eating behaviour in the immediate post-exercise period. Nevertheless, with the provision of standardised meals across conditions, both LV-HIIE and CME trials would induce a short-term energy deficit and we did not observe marked alterations in subjective or hormonal appetite responses in directions that would be expected to stimulate compensatory increases in energy intake. The provision of two meal options may have introduced between-subject variability in the appetite responses but this was minimised by closely matching the meals for energy and macronutrient content and, importantly, meals were fully standardised within participants across all three trials. Finally, this was a post-hoc analysis which is likely to be underpowered; therefore, it is possible that the largely null findings may reflect a type 2 error and require future replication before definitive conclusions can be drawn.

In conclusion, single bouts of LV-HIIE and CME increased circulating total GLP-1 concentrations but did not influence overall appetite perceptions or circulating total PYY and AG concentrations in white Europeans and South Asians with non-diabetic hyperglycaemia. Exercise-related appetite responses were not strongly influenced by exercise intensity or ethnicity. Future studies should seek to extend these comparisons to those diagnosed with cardiometabolic conditions and explore the longer-term impact of CME and LV-HIIE on appetite and weight control.

## Data Availability

Data generated in this analysis are available from the corresponding author upon reasonable request.
