# Correction: The acute effects of moderate-intensity continuous or high-intensity interval exercise on appetite and appetite-related hormones in South Asian and white European adults with non-diabetic hyperglycaemia

**DOI:** 10.1038/s41430-025-01650-w

**Published:** 2025-07-24

**Authors:** Tonghui Shen, Alice E. Thackray, Jack A. Sargeant, Thomas Yates, James A. King, Scott A. Willis, David J. Stensel

**Affiliations:** 1 Department of Physical Education and Aesthetic Education, Hangzhou City University, Zhejiang, China; 2 National Centre for Sport and Exercise Medicine, School of Sport, Exercise and Health Sciences, Loughborough University, Loughborough, United Kingdom; 3 National Institute for Health and Care Research (NIHR) Leicester Biomedical Research Centre, University Hospitals of Leicester NHS Trust and University of Leicester, Leicester, United Kingdom; 4 Diabetes Research Centre, University of Leicester, Leicester, United Kingdom; 5 Faculty of Sport Sciences, Waseda University, Tokorozawa, Japan; 6 Department of Sports Science and Physical Education, The Chinese University of Hong Kong, Hong Kong, China

**Keywords:** Risk factors, Weight management, Biomarkers

Correction to: *European Journal of Clinical Nutrition* 10.1038/s41430-025-01633-x; published online 27 May 2025

In this article, the affiliation details for Tonghui Shen were incorrectly given as ‘Department of Physical Education and Aesthetic Education, Zhejiang City University, Zhejiang, China’ but should have been ‘Department of Physical Education and Aesthetic Education, Hangzhou City University, Zhejiang, China’. The original article has been corrected.

